# Main implications related to the switch to *BRCA*1/2 tumor testing in ovarian cancer patients: a proposal of a consensus

**DOI:** 10.18632/oncotarget.24728

**Published:** 2018-04-13

**Authors:** Ettore Capoluongo, Giovanni Scambia, Jean-Marc Nabholtz

**Affiliations:** ^1^ Laboratory of Advanced Molecular Diagnostics (DIMA), Istituto Dermopatico dell’Immacolata, Fondazione Luigi Maria Monti, IRCCS, Rome, Italy; ^2^ Catholic University of The Sacred Heart, Rome, Italy; ^3^ Molipharma Srl, a Spinoff of Catholic University, Campobasso, Italy; ^4^ Cancer Research Oncology Centre, King Saud University Medical City, Riyadh, KSA

**Keywords:** OvCa, tBRCA1/2, NGS, molecular diagnostics

## Abstract

**Background:**

Since the approval of the first poly (adenosine diphosphate [ADP]) ribose polymerase inhibitor (PARPi; olaparib [Lynparza™]) for platinum-sensitive relapsed high grade ovarian cancer, with either germline or somatic *BRCA1/2* deleterious variants, the strategies for *BRCA1/2* are dynamically changing. Along with germline testing within the context of familial or sporadic ovarian cancer, patients are now being referred for *BRCA1/2* genetic assay above all for treatment decisions: in this setting tumour BRCA assay can allow to identify an estimated 3–9% of patients with peculiar somatic *BRCA1/2* mutations. These women could also benefit from PARPi therapy. This new type of approach is really challenging, in particular due to the technical and analytical difficulties regarding low quality DNA deriving from formalin-fixed, paraffin-embedded (FFPE) specimens.

**Aim:**

in this manuscript, we try to a) underline many issues related to *BRCA1/2* analysis by next generation sequencing technologies (NGS), b) provide some responses to many questions regarding this new paradigm related to OvCa patients’ management. Some considerations for incorporating genetic analysis of ovarian tumour samples into the patient pathway and ethical requirements are also provided.

**Methods:**

we used our retrospective data based on thousands of ovarian cancer women sequenced for *BRCA1/2* genes.

**Discussion:**

tumor *BRCA1/2* assay should be rapidly introduced in routine laboratory practice as first line testing by using harmonized pipelines based on consensus guidelines.

## ISSUES AND POSSIBLE SOLUTIONS REGARDING TUMOR *BRCA1/2* ASSAY BY NEXT GENERATION SEQUENCING (NGS)

### Introduction

Tumor BRCA testing (*tBRCA*) is emerging as a powerful tool to discover and identify more mutations in high serous ovarian cancer patients which have been shown to benefit from treatment with poly ADP ribose polymerase (PARP) inhibitors [1, 2]. Somatic *BRCA1/2* pathogenic variants are reported to be present in about 7% of ovarian cancers in the first line or platinum-sensitive relapsing patients [3]. Since *BRCA1/2* testing is now needed to support treatment decisions in many countries, it is crucial to ensure robust testing [3]. In fact, despite its clinical utility, using of some NGS-based technologies, able to enrich somatic mutation from FFPE samples, still requires peculiar adjustments before being completely implemented as validated routine assays [4]. Moreover, tumor testing results as more technically challenging than the germline one: however, *tBRCA* assay shows the advantage that both germline and somatic variants can be identified in a single sample [5].

Since some literature evidences are still underlying the importance of using of tumor materials as starting sample for *tBRCA1/2* profiling, we decided to progressively switch from *BRCA1/2* germline to tumor analysis [3, 5, 6]. In the last twelve months, by working with NGS technologies and pipelines set for this purpose, we experienced as the know-how and layout achieved on germline BRCA testing cannot be completely transferred to the tumor analysis, the two conditions being very different. In light to better underline the main pitfalls and criticisms regarding *tBRCA1/2* testing, it would be really important to point out and respond to the following questions before deciding to definitively switch to tumor evaluation.

### Why tumor *BRCA1/2* testing is important?

Since Hennessy et al [7] identified OvCa patients with somatic BRCA pathogenic variants, many authors have underlined as more women may take advantage from treatment with the *PARPi* (Olaparib). The latter [8] is in fact currently approved for OvCa patients carrying germline BRCA pathogenic variants (PVs) in the USA while, in the European countries and most of the world, for women with both germline and somatic BRCA mutations. Anyhow, we believe a lot of the positive impact that might result from the introduction of the *BRCA1/2* tumor testing, particularly because more patients should benefit from anti-PARP-1 targeted therapies or other similar new drugs coming soon [9, 10].

We underline as tumor genomic assays are able to identify: a) somatic (tumor-associated) variants with potential diagnostic, prognostic, and predictive therapeutic implications; b) germline pathogenic variations, with clinical implications for both patients and their family members [1, 11, 12]. Therefore, women will really benefit from this diagnostic workflow, both in terms of targeted therapy and in the way to reduce the family cancer risk, particularly of the future women generations [12].

Tumor *BRCA1/2* testing is important also as possible further surrogate biomarker of response to immune check-point inhibitors. In fact, we could hypothesize that, given that *BRCA1/2*-mutated high grade serous ovarian cancers (HGSOCs) exhibit a higher mutational load and a unique mutational signature, with an elevated number of larger indels up to 50 bp, these tumors may also harbor more tumor-specific neoantigens [13]. Therefore, they can show higher immunogenicity and survival, suggesting that *BRCA1/2*-mutated HGSOCs may be more sensitive to PD-1/PD-L1 inhibitors compared to homologous recombination (HR)-proficient HGSOCs [13].

### Are there pitfalls that can strongly affect *tBRCA* testing?

Experience and the right choice of pipeline are really crucial, as also verified in our last three hundred FFPE OvCa samples analyzed within our molecular diagnostic laboratory. Issues and pitfalls regarding the pre-analytical, analytical and post-analytical phases, surrounding any somatic NGS-based testing, have not been completely solved. For example, DNA modifications determined by low quality procedures of tissue fixation can generate lots of C:G to T:A transitions that can affect quality of variant calling. These base changes can result higher than the maximum acceptable (10%) in the hotspot cancer panel assay [14]. Unfortunately, no definitive cutoffs are reported regarding the untargeted analysis, as in the case of *tBRCA1/2* testing. Furthermore, as recently reported [3], all laboratories select their testing methods for different reasons: some are using amplicons-based approaches, some commercial kits because no assay development is required, above all when this kit fit with CE-IVD requirements for use on FFPE DNA [3].

Therefore, the definition of univocal standards and cutoff for variant calling are generally dependent on the NGS pipeline layout. Nevertheless, many platforms, chemistries or pipelines showed either general or specific pitfalls regarding germline *BRCA1/2* testing: these issues can become even more critical when the analysis is directly performed on FFPE tumor samples [6]. In fact, using Ion torrent PGM machine, Suryavanshi M et al. [15] falsely identified the *BRCA1* c.950_951 insA (p.Asn319fs) and *BRCA2* c.1032_1033 insA (p.Asn346fs) pathogenic variants which were not confirmed by Sanger sequencing. Moreover, other groups showed as 100% overlapping results was not obtained by different labs working on the same FFPE tumor samples [3]. These findings are perfectly in agreement with our experience. In fact, the same errors in variant calling can affect other peculiar variants of *BRCA1*, such as the exon16 c.4964_4982del19 (p.Ser1655Tyrfs). In the context of a retrospective study, approved by our Institution at Catholic University of Rome (Molipharma Grant n. ESR-14-10185), we used a specific pipeline run on Ion5S chemistry (Oncomine BRCA pipeline, Thermo Fisher). During the set-up of somatic NGS pipeline we used tissues belonging to patients previously screened for germline *BRCA1/2* variants, in order to verify the performances of entire pipeline (data not shown being this study under submission elsewhere). Although all the instructions provided within the datasheet were followed to achieve the best setting, the c.4964_4982del19 resulted as missing in 5/5 (100%) OvCa tissues belonging to five women previously identified as del19 germline carriers. Contrastingly, by analyzing the raw data of the NGS run performed, we verified that, although the region of interest was abundantly covered by the primers, the specific bioinformatic tool dramatically failed in the variant calling settings. Our main concerns regarding these redundant false negative results are related to the following consideration: *BRCA1* c.4964_4982del19 is very common in Italian women belonging to Calabria and Sicily regions (where it is considered as founder). Therefore, we cannot even imagine the dramatic impact of this type of pitfalls on peculiar population cohorts where some founder PVs are present. In particular, we underline as thousands of Calabrian and Sicilian women are living not only in other Italian regions but also worldwide. Nevertheless, our capability to tightly collaborate with bioinformatic support team of the Company, allowed us to fix and solve this problem within one month.

Noteworthy, in the absence of evidence of this type of pitfalls, no one would have noticed. In fact, our preliminary validation step, based on the use of specifically germline mutated DNA samples, allowed the discovering of this “bioinformatics bug” related to the Oncomine pipeline. Therefore, each new NGS pipeline should be subjected to a sort of “stress test”, using peculiar variants belonging to clinical samples along with commercially available reference and certified materials, before being introduced in routine settings. The validation set should be appropriately chosen in order to provide unequivocal data regarding robustness of each NGS *tBRCA* pipeline.

### NGS technology needs to be complemented by other Dx tools

Based on the above considerations, the proficient collaboration between Diagnostic Companions and Molecular Diagnostic Laboratory should be strongly encouraged in order to solve all these issues. The FDA definition of companion diagnostics only encompasses two types of companion diagnostics - theranostics and monitoring tests [16]. Although many companion diagnostic kits are already in use in oncology [17], drug companions should strongly take into account as NGS testing may be error prone [18–20] particularly when addressed to BRCA analysis, where the presence of large rearrangements cannot be correctly identified or predicted [21]. Our data on more than 5,500 OvCa patients tested for *gBRCA1/2*, showed as about 8% of women are carrying small or large germline rearrangements: some of these were preliminary predicted by our NGS bioinformatics algorithms and subsequently confirmed by MLPA/MAQ assays [22]. We also identified four new large deletions, a complete duplication in *BRCA1* and a complete deletion in *BRCA2* genes [21, 22]. Some reports have shown as large rearrangements can be also evaluated in more deepen through comparative genomic hybridization (CGH) array. The latter is able to verify he extension of LGRs, although the exact breakpoints of the duplication insertion should be searched in detail by other methods [23]. The remaining ones were indeed identified only after having considered family or personal clinical history. Therefore, regarding both tumor and germline *BRCA1/2* testing, we can agree as NGS alone is not enough: in fact, many strategies should be used to approach to BRCA testing [6, 21], particularly due to the complexity of these two genes along with their ability to rearrange. Therefore, NGS data, alone, cannot be completely informative for both patients and physicians managing OvCa patients.

### How to solve these issues and accelerate the routine use of *tBRCA1/2* testing?

Taking into account the above mentioned pros and cons regarding somatic *BRCA1/2* NGS testing [15, 16, 22], drug companions [16, 17] should tightly collaborate with highly skilled molecular diagnostic laboratories in order to achieve the complete harmonization of NGS pipelines used to this purpose.

## CONSENSUS PAPER

We underline as, although the recently published recommendations [3, 6, 12, 24, 25] can be considered as a useful staring point, above all for the target NGS analysis, the harmonization of *tBRCA* testing should be strongly encouraged.

It would be really hopeful that, since some professionals who experienced with this field, have already delivered a Guidance Statement on *BRCA1/2* Tumor Testing [6], all the others should contribute by soliciting the molecular diagnostic community to license a consensus document able to respond to the following critical questions:

- Type of starting samples: fresh or FFPE tissue? And which tumor cell percentage?
- Fixation and tissues processing: which TAT and how many tissue slices?
- Type of pipelines: NGS alone or NGS coupled to MLPA?
- Chemistry: target enrichment or PCR based methods?
- Minimum acceptable coverage: unique or method dependent?
- Gene panel: *BRCA1/2* testing alone or BRCA coupled to HRD in case of *BRCA1/2* negative results?
- Type of variants: how to undoubtedly discriminate the germline from somatic variants?
- Internal and external quality controls: commercial, homemade or referred to international frameworks?

To partially respond to the first question (tumor *versus* germline), it would be interesting to underline the results coming from our recent experience [26], particularly on fresh OvCa tissues. In fact, by comparing results obtained in three different reference Laboratories on the same DNA samples, we found as fresh tissues are really useful, being these starting materials able to provide high quality input tumor DNA for both qualitative (NGS) and quantitative (CNV) evaluation. Undoubtedly, the availability of fresh tissues is strictly related to the hospital organization, mainly depending on the close cooperation between surgeons, pathologists and molecular team. Therefore, the hospital organization could represent the only insurmountable obstacle to the using of fresh tissue for *tBRCA* testing: nevertheless, when well organized, this layout should be really encouraged. Therefore, multidisciplinary approach can result as always winning. Contrastingly, in absence of a coordinated “Cancer Woman Unit”, the use of standardized FFPE slides, is equally able to provide high quality NGS *BRCA1/2* analysis, particularly in the context of robust and validated pipelines.

### The improvements regarding *tBRCA* testing represent a moral obligation to the patients

Based on the above appraisals, *tBRCA1/2* testing should be strongly harmonized worldwide [3, 12] in order to: a) ensure best access to innovative therapies, b) identify in advance mutations that might involve the family, c) prevent the occurrence of a second cancer, d) facilitate the diagnostic process and family management [27–31]; e) improve results deriving from clinical ongoing and future trials [30]. In these regards, both germline and somatic *BRCA1/2* assays have the same dignity and value: in fact, when these molecular assays are correctly performed, they can provide clinically useful information regarding both family risk and targeted treatment [1, 4, 7]. Tumor testing, in fact, does not exclude the germline one, the latter being complementary above all to definitively exclude the presence of the same PV among other family members. This approach should be really useful also in the management of prostate cancer, where *BRCA1/2* assessment plays as a powerful emerging “biomarker” able to maximize personalized medicine protocols [32].

Therefore, taking into account that tumor testing can result more informative in terms of mutation detection rate, its introduction in clinical routine should be stimulated and encouraged. In this regard, we cannot forget as the role of molecular laboratory remains crucial in terms of quality assurance: consequently, clinicians should exclusively refer to laboratory of proven experience.

However, possible limitations regarding *tBRCA* testing may be related to the fact that current guidelines either include all high-grade ovarian carcinomas or all ovarian carcinomas, except mucinous, although there is no restriction to high-grade serous alone. In addition, reproducibility of histologic subtyping is low between non-expert pathologists [33]: high-grade endometrioid are often revised to high-grade serous upon expert pathologic review and the use of immunohistochemical panels. Therefore, limiting *g/tBRCA* testing to only those with high-grade serous carcinomas, at the moment, will miss certain patients and families.

Neverthelss, the contribution of other genes related to genome instability will be applied in the future to OvCa tumor testing. Mutations in high penetrant genes, such as TP53 and PTEN, and more frequent mutations in moderate penetrant genes, such as *CHEK2*, ATM and *PALB2* have in fact emerged and confirmed as breast and/or ovarian cancer susceptibility genes [34]. Multi-panel screening including these genes is going to be incorporated in laboratory routine workflow very soon. Moreover, is also true as the FDA and EMA do not require BRCA mutation status for all the antiPARP-1 maintenance therapy: nevertheless, *g/tBRCA1/2* screening still remains the most powerful strategy for breast and ovarian prevention [35]. The future direction and challenges for *PARPi* will be to continue to expand beyond BRCA and ovarian cancer by identifying molecular or functional signatures of response. We need to definitively assess if the durable responses in ovarian cancer can be improved and efficacy can be reached in other cancer sub-types by combining with novel targeted agents [35].

Finally, we can definitively underline as, in the next future, the isolation of *ctDNA* from blood and comprehensive sequencing of *BRCA1/2* or BRCA-related genes would provide a powerful method for interrogating the mutational status of HGSOCs that progress on therapy without the need for an invasive biopsy. As Mayor et coll published, “beside the identification of small insertion and deletion present in *ctDNA*, a comprehensive sequencing assay can detect a variety of previously known and unknown genomic alterations, including missense mutations, larger deletions, or rearrangements that could impact response to treatment” [36]. Novel strategies for treating tumors with acquired resistance to *PARPi* are in early stages of investigation, such as inhibition of *CDK12* or combinations of therapies such as *PARPi*, vorinostat, and 6-thioguanine [37].

## CONCLUSIONS

Our vision is perfectly in agreement with Ellison et al who stated as “Given all these considerations, it is important to drive standards and standardization in BRCA FFPE testing, particularly when the test results dictate clinical decisions regarding life extending therapies” [3]. Therefore, the road towards the development of a consensus guideline on the *BRCA1/2* testing should be fully covered quickly and facilitated through close cooperation between experts in the field of ovarian cancer specialists, pathologists, molecular biologists, and clinical-molecular geneticists. A complete multidisciplinary team approach is never like now really welcome.
